# Tubular Scaffold with Shape Recovery Effect for Cell Guide Applications

**DOI:** 10.3390/jfb6030564

**Published:** 2015-07-10

**Authors:** Kazi M. Zakir Hossain, Chenkai Zhu, Reda M. Felfel, Nusrat Sharmin, Ifty Ahmed

**Affiliations:** 1 Division of Materials, Mechanics and Structures, Faculty of Engineering, University of Nottingham, University Park, Nottingham NG7 2RD, UK; E-Mails: eaxcz4@nottingham.ac.uk (C.Z.); reda.felfel@nottingham.ac.uk (R.M.F.); eaxns1@nottingham.ac.uk (N.S.); 2 Physics Department, Faculty of Science, Mansoura University, Mansoura 35516, Egypt

**Keywords:** tubular scaffolds, PLA fibre, compressive modulus, shape recovery, cell proliferation

## Abstract

Tubular scaffolds with aligned polylactic acid (PLA) fibres were fabricated for cell guide applications by immersing rolled PLA fibre mats into a polyvinyl acetate (PVAc) solution to bind the mats. The PVAc solution was also mixed with up to 30 wt % β-tricalcium phosphate (β-TCP) content. Cross-sectional images of the scaffold materials obtained via scanning electron microscopy (SEM) revealed the aligned fibre morphology along with a significant number of voids in between the bundles of fibres. The addition of β-TCP into the scaffolds played an important role in increasing the void content from 17.1% to 25.3% for the 30 wt % β-TCP loading, which was measured via micro-CT (µCT) analysis. Furthermore, µCT analyses revealed the distribution of aggregated β-TCP particles in between the various PLA fibre layers of the scaffold. The compressive modulus properties of the scaffolds increased from 66 MPa to 83 MPa and the compressive strength properties decreased from 67 MPa to 41 MPa for the 30 wt % β-TCP content scaffold. The scaffolds produced were observed to change into a soft and flexible form which demonstrated shape recovery properties after immersion in phosphate buffered saline (PBS) media at 37 °C for 24 h. The cytocompatibility studies (using MG-63 human osteosarcoma cell line) revealed preferential cell proliferation along the longitudinal direction of the fibres as compared to the control tissue culture plastic. The manufacturing process highlighted above reveals a simple process for inducing controlled cell alignment and varying porosity features within tubular scaffolds for potential tissue engineering applications.

## 1. Introduction

Porous scaffolds can play an important role in the regeneration of host tissue affected either by disease or trauma. These scaffold materials should ideally be biocompatible, biodegradable, bioactive and also possess sufficient mechanical properties in order to maintain their three-dimensional integrity during the tissue healing process. Numerous bioresorbable materials, such as natural [1,2] and synthetic polymers [3,4], bioactive ceramics [5] and glasses [6,7,8] have been investigated extensively to fabricate solid foams [9,10,11], tubular foams [12,13,14,15] and oriented fibrous scaffolds [16,17,18] for tissue engineering applications. Porosity, geometry and degradation rate of the scaffolds should be tuneable to maximise cell attachment, proliferation, differentiation, vascularisation, tissue remodelling and production of extracellular matrix (ECM) depending on the end applications.

Tubular shaped scaffolds can possess advantageous features over solid foam type scaffolds such as guiding or encouraging cells along a specific route or to a target location. Tubular scaffolds have been manufactured from various types of biopolymers such as, polylactic acid (PLA), poly(ε-caprolactone) (PCL), poly(DL-lactic-co-glycolic acid)(PLGA) and collagen [19,20,21,22] to promote cell and/or tissue regeneration and have been investigated as nerve guides [23,24,25,26] and for tendon [27], cartilage [28,29] or bone [30,31] repair applications. The shape of the tubular scaffolds has generally been fabricated via freeze drying [26] and/or electrospinning processes [32,33,34]. For example, Jeong *et al.* [15] investigated manufacture of tubular scaffolds made using PCL with controlled fibre orientation (random, parallel or orthogonal) which were fabricated via an electrospinning process. A conductive rotating drum equipped with parallel and orthogonal wires was used to control the alignment of the fibres. The Young’s modulus and tensile strengths of these fibrous tubular scaffolds were reported to be in the range of 3 to 5 MPa and 1.7 to 3 MPa, respectively. They also demonstrated that NIH-3T3 fibroblast cells began to spread on the random-fibrous samples after 72 h of cell culture, whereas most of the cells cultured on the aligned fibrous samples stretched along the fibre direction after only 24 h of cell culture [15]. Guarino *et al.* [31] prepared a porous tubular scaffold formed by integrating poly(l-Lactide) (PLLA) fibres using a blend of NaCl salt and PCL matrix (9:1 ratio) via a filament winding process. Approximately 80% porosity was achieved within the scaffold by means of this salt leaching technique after the winding process. They also reported that no significant weight change was observed after 35 days of degradation in PBS media. They also observed that the aligned fibrous structure promoted oriented migration of bone cells (human marrow stromal cells and trabecular osteoblasts) along their direction.

Majority of the tubular scaffolds investigated in the literature have focused on nerve guide applications [35,36,37]. Den Dunne *et al.* [35] investigated biodegradable poly(D,L-lactide-ɛ-caprolactone) based conduit (12 mm length) and compared it with an autologus nerve graft to repair a nerve gap of 10 mm in a rat model. They reported that after 10 weeks the regenerated nerve contained twice the number of myelinated nerve. Whilst, for the autologous nerve graft the number of myelinated nerve fibres increased by one-third of the number in the control nerve. This suggested that nerve regeneration through this type of polymer based nerve conduit could be faster and qualitatively better when compared with an autologous nerve graft. Ngo *et al.* [36] used wet-spun PLLA microfilaments to bridge a 10 mm gap lesion in the rat sciatic nerve and reported that PLLA microfilaments were able to enhance the nerve repair and regeneration across these large nerve defects. Rangappa *et al.* [37] reported that laminin-coated PLA filaments provided an improved substratum to organise Schwann cell migration and direct axonal growth *in vitro*. Widmer *et al.* [38] fabricated porous, biodegradable tubular conduits using PLGA and PLLA for guided tissue regeneration and reported that the modulus and failure strength of PLLA conduits were ~10 times higher than those of PLGA conduits (*ca*. 8 MPa and 0.1 MPa of tensile modulus and strength, respectively). Bioresorbable tubular scaffolds has also been investigated for tendon tissue engineering [27]. The functionality of ligaments or tendons depends mainly on their highly organised microstructures. Therefore, use of aligned biodegradable fibres has been considered to be beneficial for tendon repair applications.

In addition to the orientation of fibrous structures, incorporation of media-activated shape recovery property within the scaffolds intends to retain their initial structure throughout the period of implantation could be considered a promising strategy for spongy bone repair and nerve guide application as well as for the regeneration of guided tissues or organs. Herein, a new technique was introduced for fabricating tubular scaffold with phosphate buffer saline (PBS) media-activated shape recovery effect produced utilising highly oriented PLA fibrous structures. In this study PLA fibres were manufactured using a melt-drawn process and collected on a rotating drum with traverse mode in order to maintain the unidirectional alignment of the fibres. Various mixtures of polyvinyl acetate (PVAc) and tricalcium phosphate (β-TCP) with the ratios of 100/0, 85/15 and 70/30 were used to manufacture tubular structures, where PVAc acted as a binder and the β-TCP was added to improve the biocompatibility of the scaffolds. The morphological, mechanical, *in vitro* swelling and shape recovery properties of these tubular scaffolds are reported in this study. To demonstrate the cytocompatibility of these tubular scaffolds, MG63 osteosarcoma cells were cultured onto the surface of longitudinally sectioned tubular scaffolds and investigated for their initial cell attachment and spreading.

## 2. Results

### 2.1. Morphological Properties

**Figure 1:**
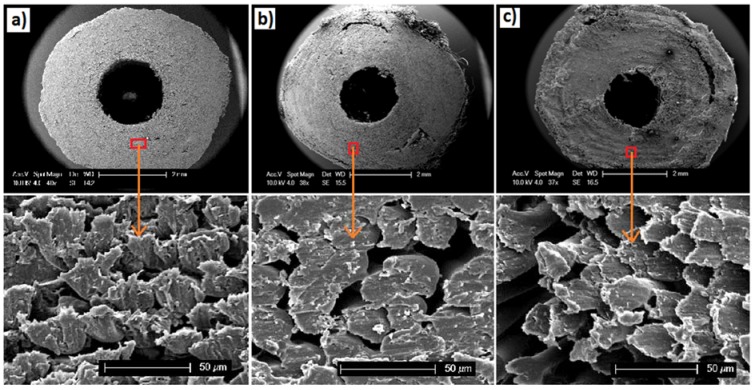
SEM images showing lateral cross sections of polylactic acid (PLA) fibre reinforced tubular scaffolds: (**a**) PLA-polyvinyl acetate (PVAc); (**b**) PLA-15% β-tricalcium phosphate (β-TCP); and (**c**) PLA-30% β-TCP. The SEM images at the bottom of each tubular scaffold reveal the void/porosity within each scaffold.

SEM images presented in Figure 1a–c revealed the lateral cross sectional morphology of the PLA fibre reinforced tubular scaffolds containing various ratios of PVAc/β-TCP blends. From Figure 1a, it can be seen that the PLA-PVAc tubular scaffold exhibited a more consolidated structure with smaller voids as compared to the other two (Figure 1b,c) PLA-PVAc/β-TCP blended scaffolds.

**Figure 2:**
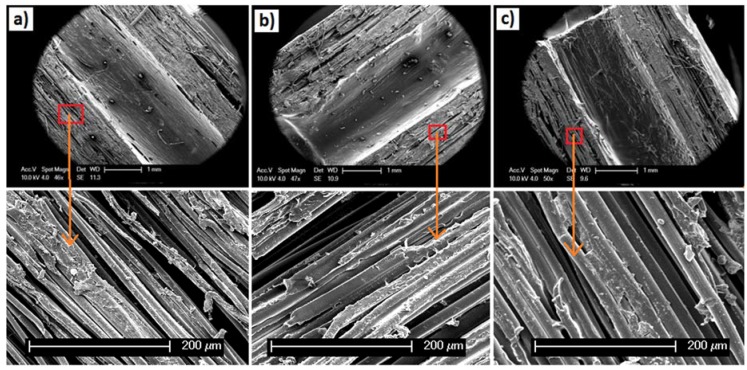
SEM images of the longitudinal cross sections of PLA fibre reinforced tubular scaffolds: (**a**) PLA-PVAc; (**b**) PLA-15% β-TCP; and (**c**) PLA-30% β-TCP. The SEM images at the bottom represent higher magnification of the scaffolds produced.

Figure 2a–c shows SEM images of the longitudinal segmental sections (cut in half) of the tubular scaffolds, where the alignment of the fibres in all of the scaffolds could be seen. In addition to this, voids in between the PLA fibres and/or the bundles of fibres were observed for all the samples.

**Figure 3:**
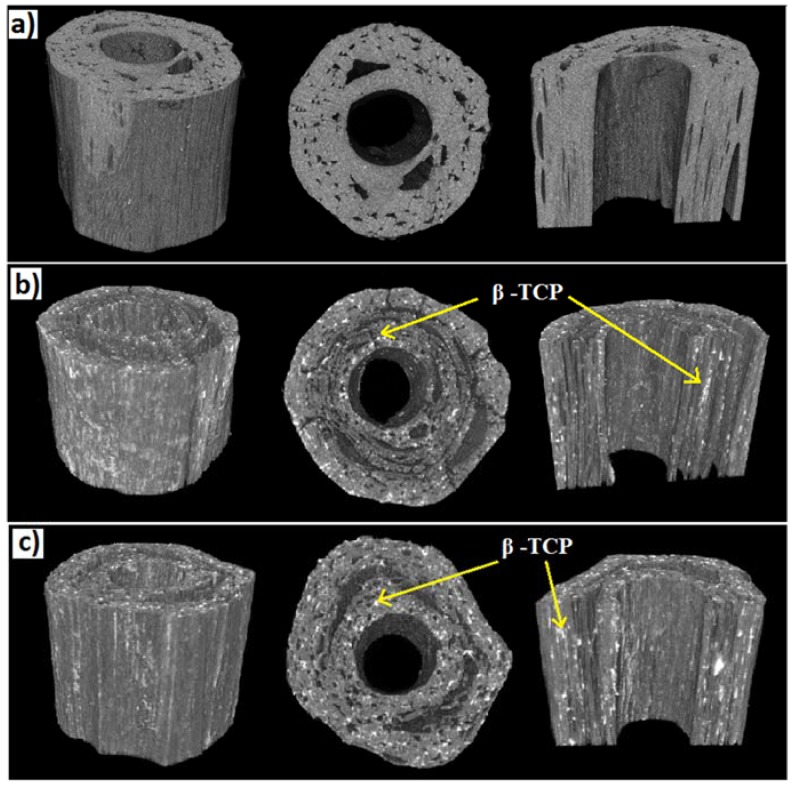
3D projections of tubular scaffolds showing the lateral cross sections and internal architectures: (**a**) PLA-PVAc; (**b**) PLA-15% β-TCP, and (**c**) PLA-30% β-TCP.

3D structures of the tubular scaffolds constructed by stacking the 2D slices obtained via micro CT scanning are presented in Figure 3. Similar to the SEM images, the 3D projections and the longitudinal segmental section (cut in half) for all scaffolds investigated showed the creation of channel-like voids within the tubes. However, compared to both types of PVAc/β-TCP scaffolds (for example, 15% and 30% β-TCP) the control PLA-PVAc scaffold did not reveal any irregular shaped white spots in the layers of the tube as observed within the detection limits of the CT system.

The percentage porosity of the tubular scaffolds was calculated from the µCT 3D data which is presented in Table 1. Approximately 17.1%, 25.0% and 25.3% porosity (excluding the large internal channel) were calculated for the PLA-PVAc, PLA-15% β-TCP, and PLA-30% β-TCP tubular scaffolds, respectively.

**Table 1:** Porosity data obtained from the micro-CT 3D data (excluding the large internal channel).

Sample	Porosity (Area %)
PLA-PVAc	17.1
PLA-15% β-TCP	25.0
PLA-30% β-TCP	25.3

### 2.2. Mechanical Properties

**Figure 4:**
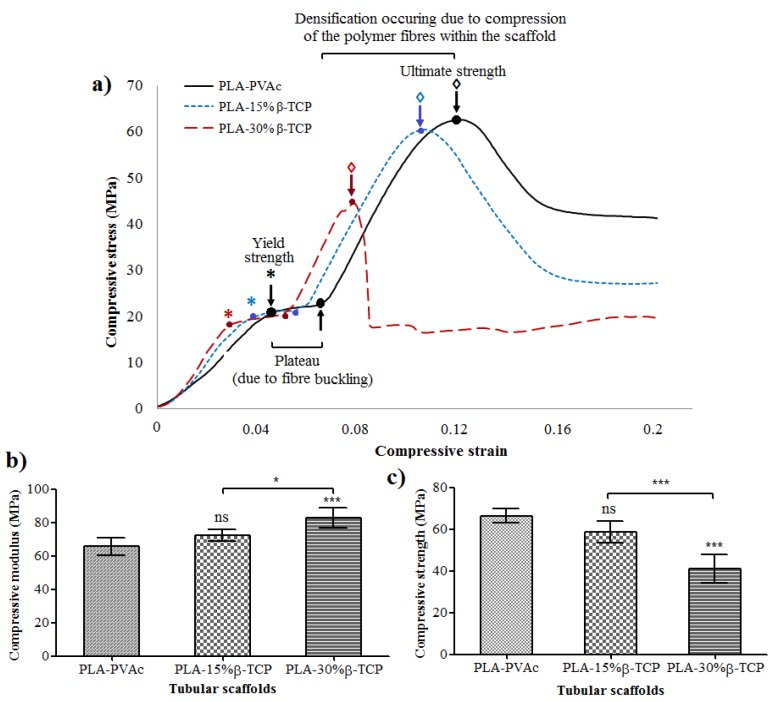
(**a**) Compressive stress-strain curves of PLA fibre reinforced tubular scaffolds; (**b**) compressive modulus and (**c**) compressive strength properties of PLA-PVAc, PLA-15% β-TCP, and PLA-30% β-TCP tubular scaffolds (ns = *p* > 0.05, * = *p* < 0.05 and *** =*p* < 0.0001).

The compressive stress-strain curve presented in Figure 4a shows the compressive properties of the tubular scaffolds produced, where the control PLA-PVAc tubular scaffold revealed a ductile profile from the compression tests. The properties of the tubular scaffolds exhibited lower compressive strength with increasing β-TCP content within the scaffolds. Compressive strength for PLA-PVAc scaffolds decreased from 67 MPa to 59 and 41 MPa by inclusion of 15% and 30% of β-TCP respectively. Moreover, it was noted that the steepness of the curves increased with the amount of β-TCP content during the initial stage of the compressive strain.

The compressive strength and modulus properties of the tubular scaffolds are presented in Figure 4b,c, where the compressive strength and modulus of PLA-PVAc tubular scaffolds were found to be 67 and 66 MPa, respectively. For the PLA/β-TCP scaffolds the compressive moduli were seen to increase with β-TCP content within the scaffolds (see Figure 4b). For example, incorporation of 15 and 30 wt % β-TCP content lead to an increase in compressive modulus properties by 9% (*p* > 0.05) and 26% (*p* = 0.0006), respectively compared to the control PLA-PVAc scaffold. However, the compressive strength properties for these scaffolds revealed a decrease with increasing β-TCP content (see Figure 4c). For example, PLA-15% β-TCP and PLA-30% β-TCP tubular scaffolds revealed a decrease in compressive strength by 12% (*p* > 0.05) and 39% (*p* < 0.0001), respectively compared to the PLA-PVAc scaffold.

### 2.3. Swelling Properties

**Figure 5:**
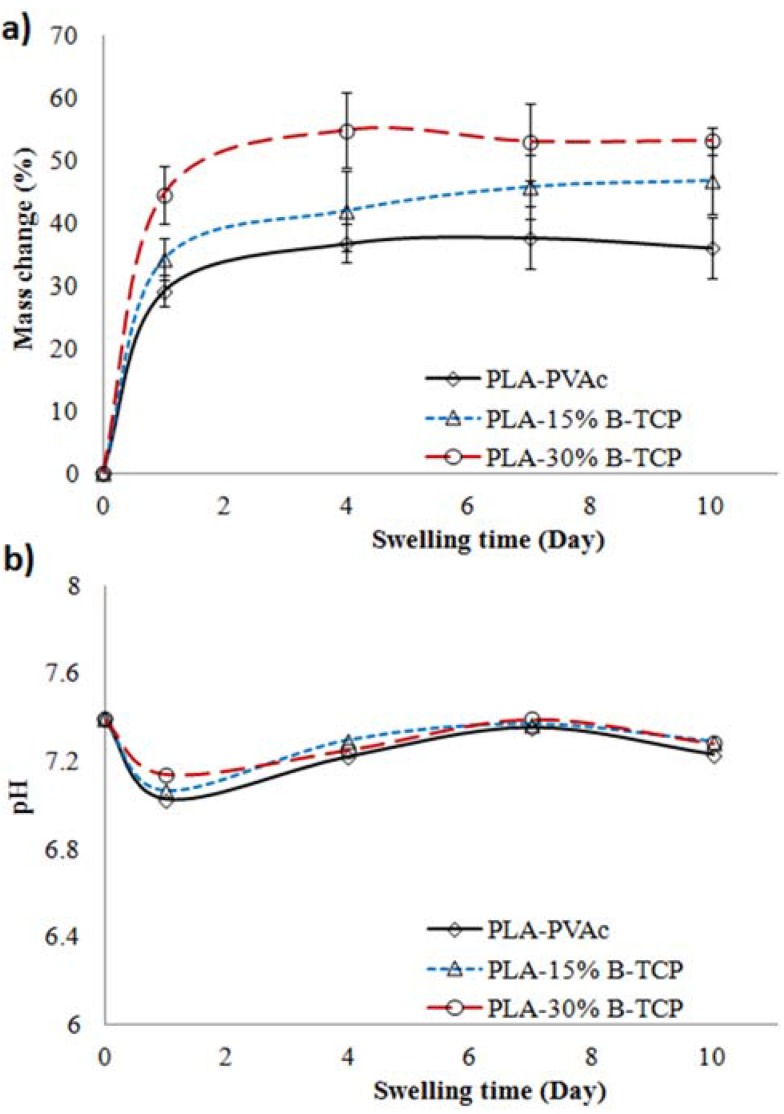
(**a**) Mass change (wet basis); and (**b**) pH of tubular scaffolds in phosphate buffered saline (PBS) media at 37 °C over 10 days of swelling period.

The percentage mass gain of the tubular scaffolds in PBS media at 37 °C over 10 days of immersion is presented in Figure 5a, where the rate of mass gain for the scaffolds was observed to increase significantly within the first 24 h. For example, PLA-30% β-TCP tubular scaffolds gained the most weight over 24 h increasing by up to 45%, whereas a 35% and 29% increase were seen for PLA-15% β-TCP and PLA-PVAc tubular scaffolds, respectively. The PLA-30% β-TCP tubular scaffolds continued to increase mass gain peaking at a 55% at Day 4 before reaching a plateau. PLA-15% β-TCP tubes showed a general trend of steadily increasing mass gain (*p* > 0.05), whereas the PLA-PVAc tubular scaffolds remained fairly constant at around a 36% mass gain over the 10 day period of the study.

The pH of the media containing the scaffolds (presented in Figure 5b) displayed a similar trend over the 10 days period. The pH for all the different types of scaffolds was seen to remain in the region of 7.2 to 7.4 over the 10 day period with the exception of Day 1, where the pH values decreased from 7.4 to around 7.1.

**Figure 6:**
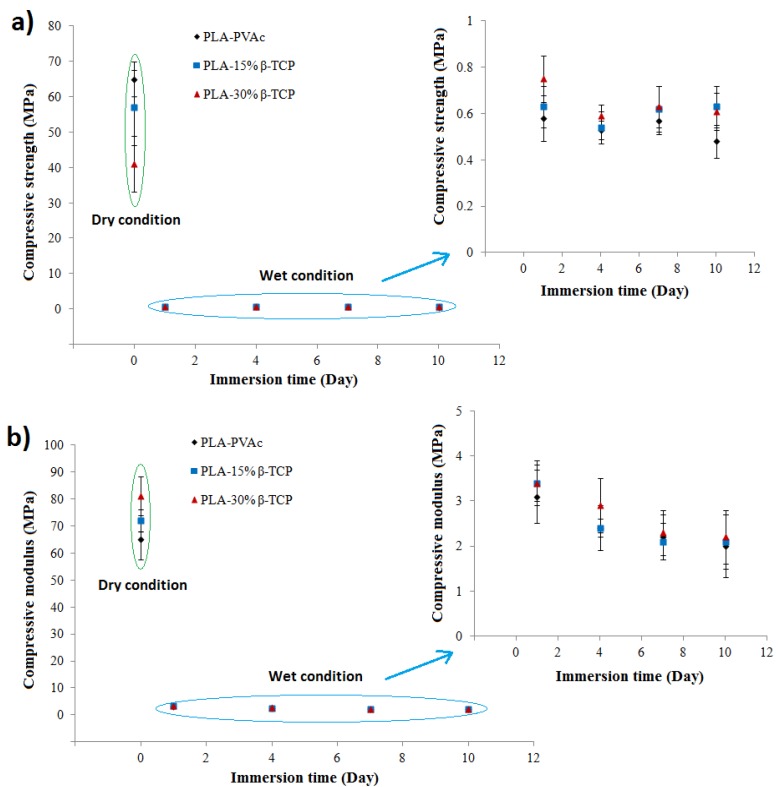
(**a**) Compressive strength; and (**b**) modulus properties of tubular scaffolds obtained after immersion in PBS media at 37 °C over the period of 10 days.

The wet compressive properties of the tubular scaffolds after swelling in PBS media over 10 days are presented in Figure 6. The compressive strength profile (Figure 6a) revealed that the maximum compressive strength of all the samples across the range of β-TCP concentrations decreased sharply after one day immersion in PBS media, and remained fairly constant up to the tenth day of immersion. Taking a closer look at the values from Day 1 to Day 10 of the study (inset of Figure 6a), it can be seen that the compressive strengths of the tubular scaffolds remained in the region of 0.50 to 0.75 MPa (*p* > 0.05). The compressive modulus of the wet tubular scaffolds revealed a decreasing trend (from 3.5 MPa to 2.2 MPa) over the 10 day immersion time (see Figure 6b), though the values were found to not be statistically significant (*p* > 0.05).

### 2.4. Shape Recovery Properties

**Figure 7:**
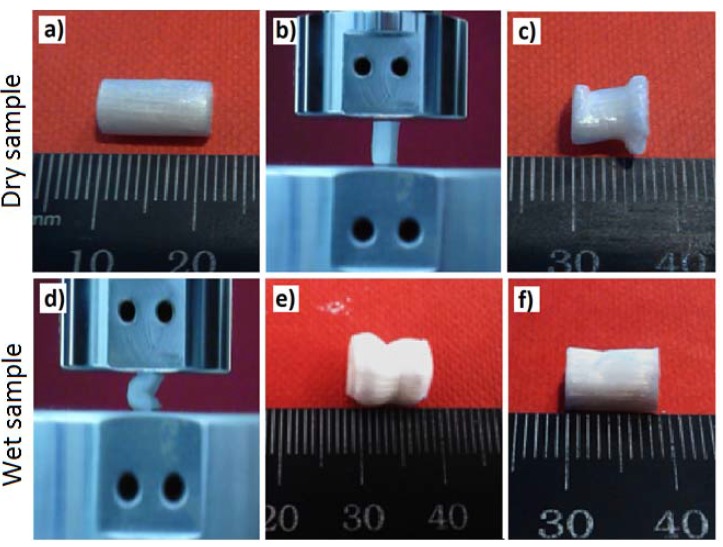
Images represent the shape recovery behaviour of the tubular scaffolds: (**a**) PLA-30% β-TCP tubular scaffolds (dry state); (**b**) setup for the compression test; (**c**) compressed PLA-30% β-TCP tubular scaffolds (dry state) showing their permanent set (image taken after three days of compression test); (**d**) compression of the wet PLA-30% β-TCP tubular scaffolds; (**e**,**f**) compressed PLA-30% β-TCP tubular scaffolds after immersion in PBS for 7 days (images were taken after (**e**) 30 s and (**f**) 5 min after compression testing).

The shape recovery properties of the tubular scaffolds manufactured displayed very similar behaviour (for both the wet and dry states) after compression testing. The images in Figure 7 represent the shape recovery behaviour of the non-immersed (*i.e.*, dry) and wet PLA-30% β-TCP tubular scaffolds (7 days in PBS at 37 °C).

The non-immersed (dry) scaffolds exhibited permanent deformation *i.e.*, did not show any evidence of recovery of their initial shape even after three days of compression test (see Figure 7c). Compared to the compressed tubular scaffolds in the dry state, all the wet scaffolds revealed shape recovery behaviour within 5 min after releasing the compression load as can be seen in Figure 7f (also see electronic supplementary information for the video).

### 2.5. Cell Study

**Figure 8:**
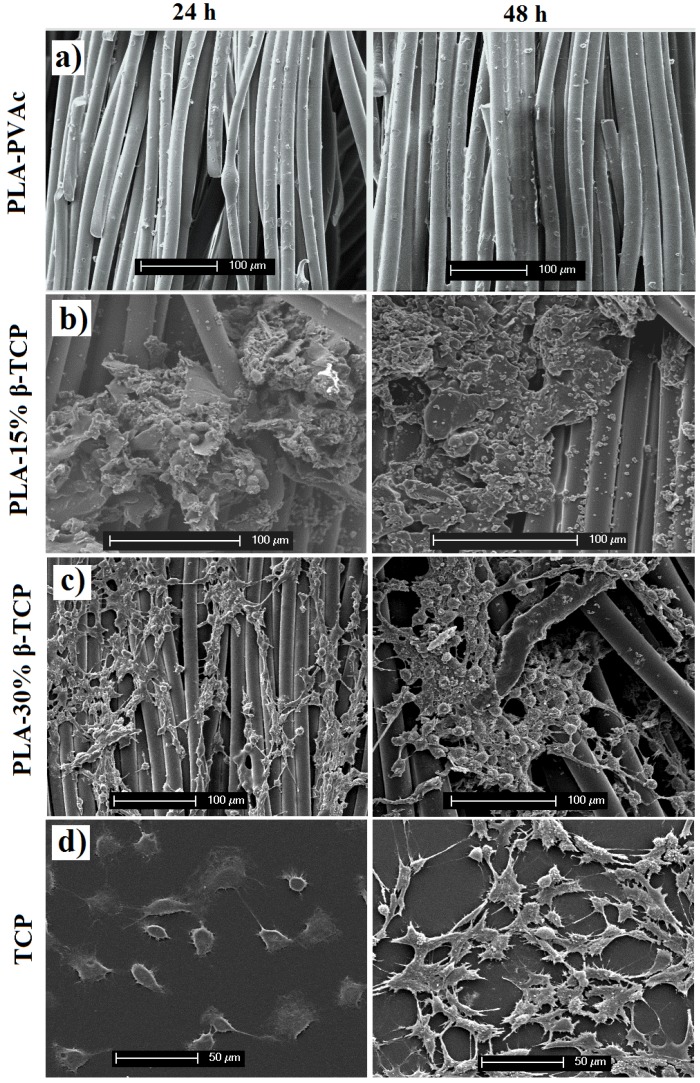
Influence of PVAc/β-TCP coating materials and fibrous structure on cultured MG-63 osteosarcoma cells morphology and spreading at varying time points (24 and 48 h): (**a**) control PLA-PVAc; (**b**) PLA 15% β-TCP; (**c**) PLA 30% β-TCP, and (**d**) control tissue culture plastic.

In this study, a preliminary short-term cell culture study was conducted for the produced tubular scaffolds. The scaffolds were seeded with MG-63 and cellular attachment monitored up to 48 h post-seeding (see Figure 8). Cell attachment was qualitatively assessed using SEM micrographs. The SEM micrographs of the MG-63 osteosarcoma cells cultured on the cross-sectional surfaces of the tubular scaffold materials are presented in Figure 8. No viable cells could be seen on the fibrous surfaces of PLA-PVAc scaffolds after seeding (as shown in Figure 8a). It was revealed that cell attachment improved with increasing amount of β-TCP. For example, the tubular scaffold containing 15 wt % β-TCP showed some evidence of MG-63 osteosarcoma cells spreading after 24 and 48 h of incubation (see Figure 8b) and the blend containing 30 wt % β-TCP supported the most promising cell spreading on the PLA fibres after 24 and 48 h of incubation as can be seen in Figure 8c.

## 3. Discussion

In this study PVAc/β-TCP blends with varying amounts of β-TCP content were used to bind aligned PLA fibres to produce tubular scaffolds. The influence of PVAc/β-TCP blends on the morphological, mechanical, shape recovery and cell attachment properties of the scaffold materials were investigated *in vitro*.

The lateral cross sectional morphology (as presented in Figure 1a–c) and the longitudinal segmental sections (Figure 2a–c) of the tubular scaffolds containing various ratios of PVAc/β-TCP blends revealed a consolidated structure of aligned PLA fibres. The PVAc applied as a binder here maintained the integrity of the PLA fibres within the tubular structures and assisted in attaching the β-TCP particles to the surface of the PLA fibres. In a previous study melt drawn PLA fibres (coated with PVAc and non-coated) with diameters ranging from 10 µm to 16 µm were reported in the literature [39,40], where PVAc was used to bind cotton based cellulose nanowhiskers (CNWs) to the surface of the PLA fibre intended to improve their biocompatibility. In this study, after consolidation of the PLA fibres into tubular shapes, the lateral cross section of the scaffold materials revealed alignment of the fibres along with a significant number of voids in between the fibres as seen in Figure 1a–c. Voids within scaffold materials have been shown to be vital for cell spreading as well as allowing for sufficient spaces for media and/or nutrients to flow within the scaffolds when conditioned in physiological environment [24]. A more consolidated fibrous structure with smaller voids was observed for the control PLA-PVAc tubular scaffold, which was due to the binding solution in between the fibres. On the other hand the PVAc/β-TCP blends in the form of a suspension allowed deposition of aggregated β-TCP particles within the scaffold; this was somewhat restricted to slower and non-homogeneous diffusion of the binding matrix in between the fibre spaces. The deposited β-TCP particles and comparatively less amount of binding materials were suggested to influence the formation of larger numbers of voids within the PVAc/β-TCP blends. Moreover, the voids in the tubular scaffolds appeared to be aligned along the longitudinal direction of the fibres which has been suggested to be favourable for guided cell proliferation [24]. Electrospun PCL fibre based multi-channels were investigated for peripheral nerve repair applications by Jeffries *et al.* [41] who observed infiltration of Schwann cells into the interior of the micro-channels.

The µCT analyses revealed deposition of aggregated β-TCP particles within the scaffolds (see Figure 3b,c) observed as white spots within the layers of the PVAc/β-TCP scaffolds. However, no white spots were observed for the PLA/PVAc (as seen from Figure 3a) within the detection limits of the system used (*i.e.*, 12 × 12 × 12 µm^3^). As such, if any β-TCP particles (less than 12 × 12 × 12 µm^3^) were present they were not detected, however the spots for Figs 3b+c were clearly evident. The β-TCP aggregates deposited within the layers of the scaffolds along with the lower amount of the binding polymer were suggested to increase the number of channel-like voids (25% porosity) observed in the different layers of the PVAc/β-TCP tubular scaffolds (see Figure 3b,c) compared to the control scaffolds (17.1% porosity).

The control PLA-PVAc tubular scaffold demonstrated ductile properties during compression testing in the dry state (see Figure 4a), which was suggested to be due to the presence of the PVAc within the scaffolds imparting a plasticisation effect, which has previously been demonstrated during the investigation of the mechanical properties of self-reinforced PLA fibre composites [41]. The yield strength of the control PLA-PVAc tubular scaffolds at approximately 20 MPa was suggested to be due to buckling of some of the aligned PLA fibres within the scaffold. The yield point was seen to further extend to a plateau (seen at about 20 MPa) which was suggested to be due to buckling of the remaining aligned fibres as well as the yield of the PVAc matrix. The ultimate strength of the PLA-PVAc scaffolds was observed at 67 MPa. Ductility can be quantified by the failure strain since failure does not commonly occur during compression testing of porous materials [42]. Therefore, either strain at yield point or at the maximum stress can be used to compute the ductility of the tubular scaffolds. By increasing β-TCP from 0 to 30%, yield strength (indicated by stars in Figure 4a) and ultimate strength (indicated by the arrows) decreased gradually which was indicative of a decrease in the scaffold ductility.

The compressive modulus of the scaffolds was seen to increase from 66 to 83 MPa via addition of β-TCP. This could have been due to the introduction of β-TCP within the plasticising layer which reduced the plasticising effect of PVAc and resulted in the enhancement of the compressive modulus properties (as seen in Figure 4b). However, the compressive strength properties of tubular scaffolds then decreased from 67 to 41 MPa with increasing β-TCP content (see Figure 4c). It is suggested that the β-TCP within the PVAc/β-TCP coating may have restricted the free flow of the PVAc in between some of the gaps amongst the fibrous structures during scaffold fabrication, which subsequently lead to the formation of voids as observed from the cross-sectional SEM and µCT images (see Figure 2b,c and Figure 3b,c). The scaffolds produced in the current study behave similarly to particulate reinforced composites. Modulus of the particulate composites generally increases with increasing filler content. Conversely, strength of the composite is more dependent on adhesion between matrix (PLA-PVAc) and filler (β-TCP) [43]. Therefore, decrease in compressive strength of the scaffolds by addition of TCP could be attributed to poor matrix-filler bonding. Similar findings were presented by Shikinami *et al.* [44] for PLA reinforced with hydroxyapatite (HA). They found that compressive modulus of PLA increased from 4.8 to 5.6 GPa by inclusion of 30% of HA, whilst the compressive strength decreased from 123 to 106 MPa. Wei *et al.* [45] also revealed that PLA-HA scaffolds showed increase in compressive moduli by increasing the amount of HA. Moreover, compressive strength and modulus of scaffolds are inversely proportional to percentage of porosity [42]. Consequently, decrease in strength for TCP reinforced scaffolds could also ascribed to increase in the porosity (see Table 1).

The mass gains observed for the scaffolds immersed in PBS media were seen to increase dramatically over 24 h. For example, PLA-PVAc tubes gained approximately 29% mass after 24 h of immersion in PBS, whereas the PLA-30%β-TCP scaffolds revealed a 45% mass gain at the same time point. This suggested that the PBS media disrupted the physical cross-linking through weakening of inter and intra-molecular hydrogen bonds within the PVAc molecules [46,47]. Xinghai *et al.* [48] reported that water absorption capacity of pure PVAc was around 32.5% after immersion for 24 h at room temperature. In addition, the higher mass gain observed for the PVAc/β-TCP scaffolds was expected due to the presence of the voids observed above (confirmed via SEM and µCT analyses). The pH for the scaffolds immersed in PBS was seen to remain relatively neutral (*i.e.*, in the region of 7.1 to 7.4) over a 10 day period. A slight decrease in pH during the early day of swelling period (*i.e.*, day 1) could be attributed to be due to the release of some unbound or loosely bound PVAc and β-TCP molecules into the PBS media.

**Figure 9:**
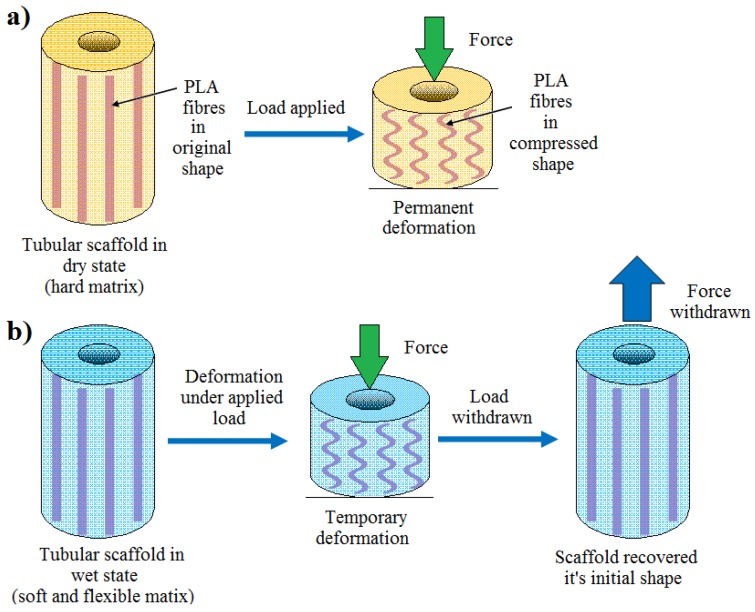
Schemes representing the tubular scaffolds undergo (**a**) a permanent deformation in dry state and (**b**) shape recovery behaviour of wet sample.

The wet compressive strength (for all the tubular scaffolds produced) revealed a significant decrease from 65 MPa to approximately 0.6 MPa after 24 h immersion in PBS media. This was suggested to be due to media absorbing the PVAc, which transformed the dry and stiff PVAc matrix into a soft and flexible matrix. Due to this plasticising property PVAc has been widely investigated for extended drug release applications [49]. Whilst application of the PVAc to the PLA fibres strongly influenced the compressive strength of the dry scaffolds, the addition of β-TCP (up to 30 wt %) in the PVAc coating only imparted a small effect on the compressive strength of the samples after immersion in PBS at 37 °C over the 10 day period. The wet compressive modulus properties were also observed to significantly decrease from 64 MPa to around 2.2 MPa due to media aided plasticising effect of PVAc described above. Therefore, all types of wet scaffolds exhibited shape recovery properties after compression test. On the other hand the hard and stiff matrix containing aligned PLA fibres failed to regain its original dimension after compression due to the permanent set (as can be seen Figure 9a). This shape recovery behaviour of wet samples was expected due to the formation of previously mentioned media aided soft and flexible matrix which didn’t show any significant effect on the mechanical properties of the wet samples, thus allowed to the recover the compressed fibres and wet matrices to its original shape when the load was withdrawn (Figure 9b). Good recovery of the scaffolds under wet conditions could be ascribed to two parameters; elasticity of swollen matrix (PVAc) and restoration of the reinforcing fibres their initial shape after release of loading. Elasticity of PVAc was attributed to plasticisation effect of water. For dry scaffolds, elastic recovery was poor as a result of water absence. Therefore, it can be drawn that wet PVAc has a dominant effect over the fibres on the elastic recovery of wet scaffolds.

Initial attachment and proliferation of MG-63 osteosarcoma cells cultured on the longitudinal cross-sectional surface of the tubular scaffolds were investigated (as presented in Figure 8). Cell attachment to PLA-PVAc scaffolds was significantly minor (as shown in Figure 8a) compared to other compositions and was attributed to the higher concentrations of PVAc [40]. Previous studies reported on NIH-3T3 mouse fibroblast cell attachment and proliferation on the surface of PVAc/Cellulose nanowhiskers coated PLA films, which suggested the similar reason (*i.e.*, higher PVAc concentrations) for not surviving the cells on the surface of PVAc coated PLA film [40]. However, it has also been suggested that decreasing the concentration of PVAc and incorporating a biocompatible material (*i.e.*, blend of 75 wt % cellulose nanowhiskers and 25 wt % PVAc) it could be possible to improve their initial cell functioning (attachment, spreading). Therefore, in this study two different weight percentages of a well-known bioactive material (15 and 30 wt % β-TCP) was added to the PVAc to investigate initial cell functions. The tubular scaffold containing 15 wt % β-TCP showed some evidence of MG-63 osteosarcoma cell spreading after 24 and 48 h of incubation (see Figure 8b) though they didn’t appear to look healthy. However, the PLA-30% β-TCP scaffolds revealed strong cell spreading after 24 and 48 h of incubation as seen in Figure 8c. Initial cell attachment of osteosarcoma cells on the PLA-30% β-TCP tubular scaffolds within the first 24 h revealed both flat (well-attached and spread) and rounded cells, extending their filopodia to anchor onto the fibrous surface and proliferation along the longitudinal direction of the fibre (Figure 8c). After 48 h, osteosarcoma cells were observed to be spread in between the fibre gaps, though the morphology of these cells was not as flat as on the control tissue culture plastic seen in Figure 8d. The cells on tissue culture plastic surface after culture over 24 h period were seen to spread all around where the cells seeded on the PLA-30% β-TCP tubular scaffolds appeared to proliferate along the longitudinal direction of the PLA fibre.

These initial cell functions (attachment, spreading, proliferation *etc*.) suggested that the PLA-30% β-TCP tubular scaffolds exhibited good biocompatibility with MG-63 osteosarcoma cells. Also, the spreading of the cells along the longitudinal direction of the fibres and the scaffold shape recovery behaviour *in vitro* suggested that these tubular scaffolds could potentially be used for cell guiding based tissue regeneration applications for example, in nerve or spinal cord repair applications. The proposed potential use of the devices for this type of scaffold could also be utilised for bone repair applications where maintaining the intramedullary flow was required. With regards the nerve guide application similar gelatine-TCP scaffolds/composites [50,51,52] have been investigated (both *in vitro* and *in vivo*) for nerve repair application. The advantage of using β-TCP was to enhance the biocompatibility of the construct and it has the additional benefit of being resorbable. The resorption of β-TCP could also increase the porosity within the scaffold which could also be favourable for tissue regeneration.

## 4. Experimental Section

### 4.1. PLA Fibre Drawing and Production of Fibre Mat

PLA fibres were produced via a melt-drawing process previously described elsewhere [39,40]. Briefly, PLA beads (NatureWorks LLC, Minnetonka, MN, USA, Ingeo™ Grade 3251D, average *M*_w_ ~ 90,000–120,000 g·mol^−1^, Density = 1.24 g·cm^−3^) were melted at 180 °C using a steel mould, which was heated with a band heater, and comprising a 2 mm hole at its base. Molten polymer exited the opening at the base due to gravity and was collected on a rotating drum with traverse mode enabling a 0.025 mm spacing (the drum diameter was 1 m with a collector distance of approximately 50 cm, rotating at approximately 400 m·min^−1^).

### 4.2. Preparation of Coating Materials

The coating materials were produced by blending different ratios of polyvinyl acetate (PVAc) and tri-calcium phosphate (β-TCP*)* (formulation of composition highlighted in Table 2) for 1 h at room temperature maintaining a 1 w/v% suspension in DI water. PVAc was obtained as Kollicoat SR (Sigma Aldrich, Gillingham, UK), a 30% dispersion of PVAc (weight average M_w_ ~ 450,000 density 1.045 g·cm^−3^ and viscosity ~100 mPa.s at 20 °C) and β-TCP were purchased from Sigma Aldrich.

**Table 2:** Formulations of coating materials prepared using PVAc and β-TCP for PLA fibre mats production.

Sample Codes Used in This Study	Composition of Coating Blends				Overall Coating Materials Deposited within the Tubular Scaffolds (Obtained after Drying the Tubes at 37 °C for 48 h) (wt %)
	PVAc (g)	β-TCP (g)	Deionised Water (mL)	Concentration of β-TCP in the Coating Suspension (wt %)	
PLA PVAc	1	–	100	0	28 ± 2
PLA-15% β-TCP	0.85	0.15	100	15	27 ± 1
PLA-30% β-TCP	0.70	0.30	100	30	29 ± 2

### 4.3. Manufacture of PLA Fibre Mats and Tubular Scaffolds

**Figure 10:**
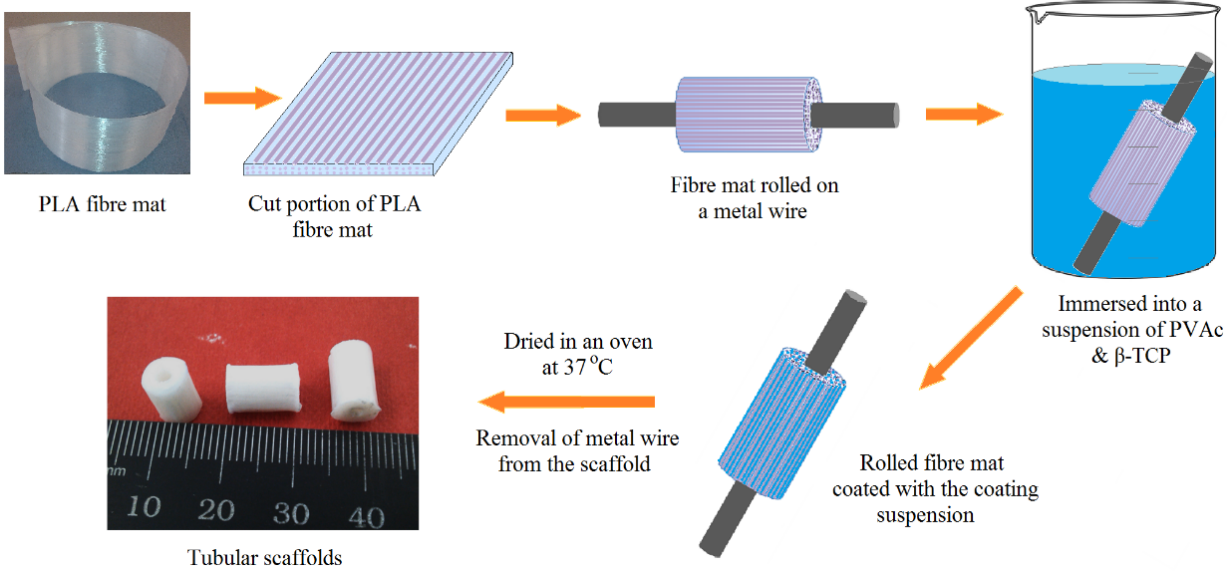
Scheme of the processing route of aligned PLA fibre reinforced tubular scaffolds.

Aligned PLA fibre mats (consisting of 10 layers of PLA fibres) were produced and the coating materials (highlighted in Table 2) were applied using a syringe needle followed by gently brushing to disperse the coating materials [41]. Fibre mats (with an average thickness of 0.4 mm containing around 1.2 wt % of coating materials) were prepared and removed from the drum after drying at room temperature for 16 h. These PLA fibre mats (40 mm × 80 mm dimension) were then wrapped manually around a metal wire (diameter 2.38 mm) and then dipped into the coating blends for 30 min before being dried at 37 °C for 48 h to produce tubular scaffolds as presented in Figure 10.

### 4.4. Characterisation

#### 4.4.1. Scanning Electron Microscopic (SEM) Analysis

The lateral and longitudinal morphology from sample cross sections of the tubular scaffolds were characterised using a Philips XL30 (FEI, North Billerica, MA, USA) SEM operated at an accelerating voltage of 10 kV. A sputtered platinum coating was employed for electron microscopic analysis to avoid image distortion due to charging.

#### 4.4.2. Microcomputed Tomography (μCT)

Tubular scaffolds (height of 10 mm, outer diameter 6 mm and internal diameter 2.38 mm) were placed in the sample holder and scanned under the micro CT (µCT40, Scanco Medical, Brüttisellen, Switzerland). A medium-resolution of 12 µm with X-ray source voltage of 70 kV and beam current of 114 µA was used for scanning the tubular scaffolds in order to acquire each tomogram of 1024 × 1024 pixels. The µCT 2D images acquired directly from the scan were further processed with optimised thresholds and analysed using ImageJ software (version 1.49n, the National Institute of Health, Bethesda, MD, USA) to construct the 3D structures. The porosity of the tubular scaffolds was calculated from the µCT 3D projection data (minus the volume of central hollow section from the total volume).

#### 4.4.3. Compression Properties

The compression properties of the tubular scaffolds (height of 10 mm, outer diameter of 6 mm and internal diameter of 2.38 mm) along their longitudinal direction were obtained until 50% strain reached using a Hounsfield compression testing machine utilising a cross head speed of 1 mm·min^−1^ and a calibrated 5 kN load cell (resolution of 0.2 N with accuracy of 0.5%). The average values of compressive strength and modulus were presented from at least five repeat specimens.

#### 4.4.4. Swelling Properties in Phosphate Buffer Saline (PBS) Media

The swelling properties of the tubular scaffold materials were calculated gravimetrically by measuring the mass of the sample before and after immersion in PBS media at 37 °C (described elsewhere [53]) and at different time intervals of 1, 4, 7 and 10 days. pH values of PBS media containing the swollen sample at varying time points (1, 4, 7 and 10 days) were also recorded and the media was replaced with fresh PBS for each sample at every time point.

Compressive mechanical properties of the swollen tubular scaffolds (in wet condition) were measured to investigate the influence of PBS media absorption on the mechanical properties of the scaffolds.

#### 4.4.5. Cell attachment and Morphology Assessment

MG63 cells (human osteosarcoma) were obtained from the European Collection of Cell Cultures (ECACC) and cell attachment and proliferation properties were investigated on the longitudinal cross sectional surface of the scaffolds. Cells were cultured in Dulbecco’s Modified Eagle Medium (DMEM) supplemented with 8.6% foetal bovine serum (FBS), 1.7% HEPES buffer, 1.7% antibiotics-antimycotics, 0.86% glutamine, 0.86% nonessential amino acids (Gibco Invitrogen, Paisley, UK) and 0.13 g L^−1^ ascorbic acid (Sigma Aldrich). 75 cm^3^ flasks (Falcon, Becton, Dickins and company, Oxford, UK) were used in culturing the cells at 37 °C in a humidified atmosphere with 5% CO_2_. When the MG63 cells reached confluency, they were detached from the flask using trypsin-EDTA solution (HBSS containing 2.5 g·L^−1^ trypsin and 0.18 g·L^−1^ EDTA). Cells were then centrifuged for 4 min at 1200 rpm followed by re-suspension in fresh media.

The tubular scaffolds (6 mm diameter) were cut in half along the longitudinal axis and sterilised by immersing into 70% ethanol for 30 min. Samples were then inserted into 24-well plates (Fisher science, Loughborough, UK) and left under UV light for 1 h for further sterilisation prior to cell culture.Tissue culture plastic (TCP) (Thermanox coverslips, Nalge Nunc International, New York, NY, USA) was used as a positive control for cell growth. Cells were seeded onto the cross section of the scaffolds at a density of 40,000 cells cm^−2^ and incubated at 37 °C for up to 48 h at predetermined time points (24 and 48 h). After washing with warm PBS three times at 37 °C, the samples were fixed by applying a 3% glutaraldehyde in 0.1 M cacodylate buffer for 30 min. Fixed samples were then washed twice using0.2 M cacodylate buffer and covered by 1% osmium tetroxide in PBS for 45 min. Samples were dehydrated gradually using mixtures of ethanol and water from 20% to 100% ethanol in discrete 10% increments, with each step lasting 5 min. Afterwards, the samples were dried using hexamethyldisilazane (HMDS) and sputter-coated with platinum for observing via a scanning electron microscope (Philips XL30, North Billerica, MA, USA) operated at 10 kV.

#### 4.4.6. Statistical Analysis

One-way ANOVA test was conducted using GraphPad Prism software (version 5) to investigate the statistical significance between the means of the data sets obtained. *p* < 0.05 (with a 95% confidence interval) was considered to be statistically significant.

## 5. Conclusions

Tubular scaffolds were fabricated maintaining a well-aligned PLA fibrous morphology using a blend of PVAc and β-TCP, where PVAc acted as a binder and β-TCP improved the bioactivity of the scaffolds. The cross-sectional morphology of the tubular scaffolds confirmed alignment of the fibres along with a significant number of voids in between the PLA fibre layers. Furthermore, the aggregated distribution pattern of β-TCP within the various layers of the scaffolds revealed an increase in the void content from 17.1% to 25.3%. Incorporation of β-TCP particles also increased the compressive modulus properties from 66 MPa to 83 MPa. However, a decrease in the compressive strength from 67 MPa to 41 MPa with addition of 30 wt % β-TCP in the scaffolds was also seen. This was suggested to be due to the increasing number of voids created within the scaffolds. The wet scaffolds showed a mass gain of 45% after immersion in PBS for one day and revealed shape-recovery properties for the wet scaffolds post compression testing. It was also seen that the orientation of the PLA fibres and addition of β-TCP supported MG-63 human osteosarcoma cell attachment and spreading along the longitudinal direction of the fibres compared to the non- β-TCP content scaffolds. This type of tubular scaffold could potentially be utilised for specific cell guided proliferation applications for tendon/ligament repair or as potential nerve guide conduits.

## Supplementary Files

Supplementary File 1
